# Sensory panel results of a dried fish powder supplement among caregivers and young children in Zambia

**DOI:** 10.1017/S1368980023002586

**Published:** 2023-11-30

**Authors:** Kathleen Ragsdale, Mary R Read-Wahidi, Netsayi N Mudege, Lora L Iannotti, Lizzy Muzungaire, Priscilla Funduluka

**Affiliations:** 1 Social Science Research Center, Mississippi State University, PO Box 5287, Mississippi State, MS 39762, USA; 2 WorldFish, Olympia Park, Lusaka, Zambia; 3 Brown School, Institute for Public Health, Washington University in St. Louis, St. Louis, MO, USA; 4 School of Public Health and Environmental Sciences, Levy Mwanawasa Medical University, Lusaka, Zambia

**Keywords:** Complementary food for Africa+dried fish powder, Protein/micronutrient blend, Fortification, Sensory panels, Zambia

## Abstract

**Objective:**

To evaluate the acceptability of traditional Zambian dishes fortified with Complementary Food for Africa+Dried Fish Powder (ComFA+Fish), a locally sourced protein/micronutrient blend designed to impact nutrient deficiencies among infants and young children (IYC) and improve pregnancy and birth outcomes among women of reproductive age (WRA).

**Design:**

During two sensory panels, caregivers evaluated: (1) the acceptability of four ComFA+Fish dishes for household consumption, including fortified chibwabwa fisashi, savory Kapenta chutney, fortified complementary maize porridge and fortified bean-vegetable soup and (2) whether their IYC found the fortified complementary maize porridge acceptable.

**Setting:**

Lake Kariba, Southern Province, Zambia.

**Participants:**

Women of reproductive age (*n* 42) and their IYC aged 6–11 months (*n* 16) and 12–23 months (*n* 26) were recruited from fishing villages in Gwembe, Siavonga and Sinazongwe District.

**Results:**

A majority of caregivers extremely liked/liked the: (1) fortified chibwabwa fisashi’s sensory attributes (94·7 %), convenience (92·8 %) and overall acceptability (100 %); (2) savory Kapenta chutney’s sensory attributes (81·8 %), convenience (92·8 %) and overall acceptability (100 %); (3) fortified complementary porridge’s sensory attributes (83·5 %), convenience (90·5 %) and overall acceptability (88·1 %) and (4) fortified bean-vegetable soup’s sensory attributes (66·8 %), convenience (87·5 %) and overall acceptability (87·5 %). Further, a majority of caregivers evaluated the fortified complementary porridge as highly acceptable to their IYC.

**Conclusions:**

Results suggest that ComFA+Fish is strategically well placed to fill nutritional gaps among IYC and WRA in Zambia and has the potential to be scaled across sub-Saharan Africa.

Zambia and other low- and middle-income countries (LMIC) across sub-Saharan Africa are expected to see increased food insecurity and undernutrition due to long-term impacts associated with climate change and the COVID-19 pandemic^(1)^. Consequently, it is also expected that the nutritional status of children living in extreme poverty in these LMIC – which is already precarious – will likely be further negatively impacted^(2,3)^. This is of grave concern, as nutritional inequalities detrimentally affect childhood health and development and can have long-term impacts on health into adulthood^(4,5)^. For example, while chronic malnutrition in young children often manifests as stunting (height-for-age<−2), it can also have long-term effects on cognitive development, general health/ability to work and educational attainment that can impact economic productivity and health (e.g. maternal reproductive health) into adulthood^(6)^.

Although Zambia has made significant progress in reducing child malnutrition in the last two decades^(7)^, rates of stunting and wasting remain high (particularly in rural areas) and Zambia ranks 140th among 163 countries in meeting the sustainable development goals^(8,9)^. Among Zambian children under 5 years, 35 % are stunted as a consequence of chronic or recurring malnourishment and 4 % are wasted as a consequence of acute malnourishment^(7)^. The first 1000 d of life – which is defined as the critical period of cognitive and physical development from conception until a child reaches 2 years of age – is identified as a key intervention point for supplementing the diets of infants and young children and of pregnant/lactating women in LMIC, particularly in Africa where stunting prevalence is highest^(6)^. However, as Eaton *et al.* (2019) report in a recent systematic review, a majority of such interventions implemented in LMIC have been cereal-based (*v*. based on animal-source foods, including aquatic animal-source foods), and their impacts on childhood malnutrition outcomes remain ambiguous^(10)^.

Dried fish powder is an important but underutilised aquatic animal-source food to address childhood stunting and hidden hunger (i.e. micronutrient deficiencies) linked to extreme poverty, lack of dietary diversity, low consumption of protein and over-dependence on the high-phytate cereal-based diets of many vulnerable households across sub-Saharan Africa^(11–14)^. Indeed, leading international organisations (e.g. FAO of the UN, WHO, U.S. Agency for International Development) as well as leading governmental organisations within Zambia (e.g. Ministry of Health, National Food and Nutrition Commission, Ministry of Fisheries and Livestock) are increasingly promoting the use of dried fish powder to meet the nutritional needs of infants and young children and of pregnant/lactating women in low-resource households^(15–21)^. Such support is well founded, as a number of current studies have clearly documented the high nutritional value of dried fish powder made from *Kapenta* (*Limnothrissa miodon* and *Stolothrissa tanganicae*)^(17–19)^.

For example, Byrd and colleagues found that a 10 g serving of dried Kapenta powder provided 20 % or more of the recommended daily intake for Ca and 37 % or more of the recommended daily intake for DHA for 6–23-month-olds^(17)^. They also found that the nutrient density of dried Kapenta powder was similar in Fe and Zn and higher in Ca and DHA to that of a small-quantity lipid-based nutrient supplement plus (SQ-LNS-plus)^(17)^. Likewise, Haug and colleagues found that a small quantity of dried Kapenta powder met the recommended daily intakes of nitrogen, Ca, phosphorus, Mg, Fe, iodine, Se, Zn and fluoride for 12–36-month-olds^(18)^, while Nölle and colleagues found that 6–7 g (∼1·5 tsp.) of dried Kapenta powder provided the recommended daily intake of vitamin B_12_. Such findings on the nutrient density of dried Kapenta powder are important, given that the gastric capacity of infants and young children is quite limited and their meals, therefore, should be nutrient dense^(19)^. Such findings also suggest that fortifying traditional dishes with nutrient dense dried fish powder is a formidable resource for enhancing the diets of nutrient-deficient infants, young children and other household members^(20)^.

The need for a low-cost, locally sourced means by which to increase the nutrient density of traditional dishes across sub-Saharan Africa is clear given that (non-fortified) maize-based foods are highly prevalent in this region, which consumes 21 % of the maize produced worldwide^(21,22)^. Indeed, considered the national staple food of Zambia^(23)^, maize is inexpensive enough that many Zambians eat *Nshima* – a traditional dish of ground white maize meal boiled with water into a stiff smooth paste – at 2–3 of their daily meals. Nshima is also commonly used as a complementary food for infants and young children by thinning the maize paste (usually with water among poorer households) into a porridge of the desired consistency. There is a need to fortify these foods given that the non-fortified maize meal traditionally used to make Nshima and complementary maize porridge is lacking in protein and micronutrients and can reduce absorption of other nutrients.

## Complementary Food for Africa+Dried Fish Powder

Although multiple micronutrient powders – internationally standardised prepackaged sachets of vitamins and minerals produced by a handful of global suppliers and distributed by donors such as UNICEF^(24)^ – have helped reduce global rates of stunting^(25,26)^ and anaemia^(27–29)^ among vulnerable children, they have had less impact on protein malnutrition^(27–32)^. In contrast, whole pelagic small fish provide protein, fat, ∼15 essential micronutrients and vitamins A, C, B_12_, E and D even when consumed in small quantities^(17–19,33–36)^. In order to help fill both protein and micronutrient gaps among vulnerable children and other household members, we conducted two sensory panels of traditional Zambian foods fortified with a locally sourced, low-cost, high-quality protein/micronutrient blend whose primary ingredient is whole pelagic small fish that are dried and ground into a fine powder. We call this protein/micronutrient blend, *Complementary Food for Africa+Dried Fish Powder* (ComFA+Fish).

As this study was conducted at Lake Kariba, which is a major source of Kapenta, we used this fish as the primary ingredient of the ComFA+Fish protein/micronutrient blend. One of the most popular freshwater small fish in Zambia – particularly for low-resource and vulnerable households – tiny Kapenta are a nutritional powerhouse^(17–19,33–36)^ and ‘major source of fish protein for all levels of society and make up a significant part of animal protein consumed in the country’^(37,2)^. Kapenta belong to the same family as herring and sardine and because these pelagic fish are small (∼10 cm long), they are dried whole, which further concentrates their nutritional density. An appetising and affordable staple food throughout Zambia and across sub-Saharan Africa for infants, children and adults, pelagic small fish such as Kapenta, Chisense (*Potamothrissa acutirostris* and *Poecilothrissa moeruensis*) and Dagaa (*Rastrineobola argentea*)^(38,39)^ can be consumed fresh, sun dried or – as is the case with ComFA+Fish – sun dried, dry roasted and ground into a fine powder that is a rich source of protein and micronutrients. Dried fish powder is produced at the household level in small batches using a mortar and pestle and is also widely available at local markets, where it is commercially produced using a mechanised grinder (e.g. a groundnut grinder) and sold in large batches and as sachets.

As adding a supplement to a food has the potential to change the sensory properties of that food, our objectives in the present study were to: (1) develop four ComFA+Fish dishes based on traditional Zambian dishes (including fortified complementary maize porridge); (2) conduct Sensory Panel I among forty-two caregivers to evaluate whether the four ComFA+Fish dishes can be produced at the household level and are deemed acceptable for household consumption by the caregivers and (3) conduct Sensory Panel II among the same forty-two caregivers and their children aged 6–23 months in order for caregivers to evaluate their child’s global liking of the ComFA+Fish complementary maize porridge.

## Methods

### Kapenta dried fish powder: analysis of nutrient composition

**Fig. 1:**
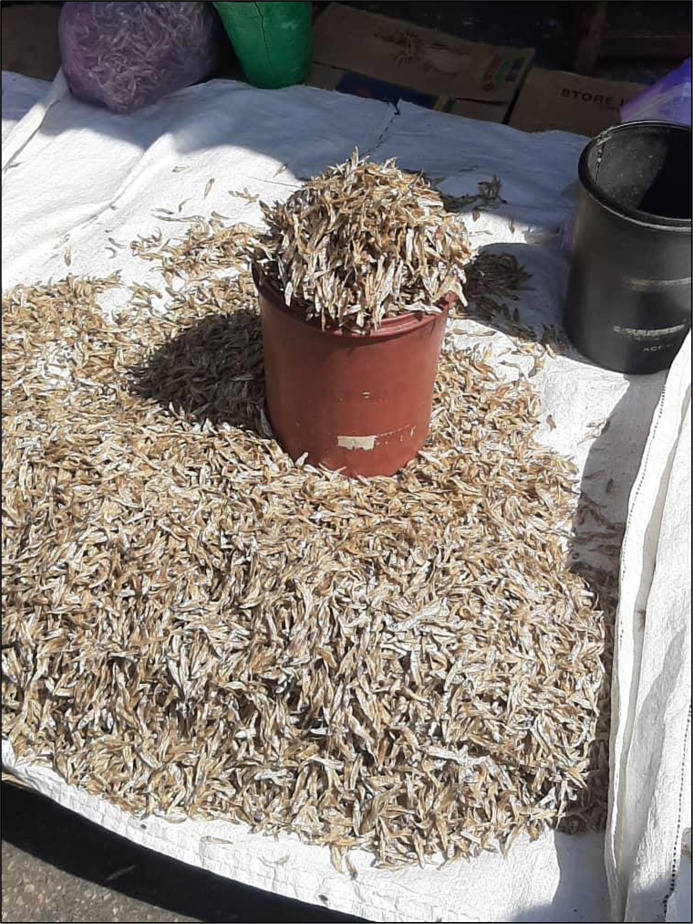
Smallscale vendor’s display of whole dried Kapenta at an open market in Lusaka, Zambia. Photo: A. Chileya, WorldFish Zambia

Following a four-step sampling protocol, we collected four individual samples of whole dried Kapenta sourced from Lake Kariba from four separate small-scale vendors at open markets in Lusaka (see Fig. 1). Each individual sample weighed a minimum of 0·5 kg, and each sample was inspected for wholesomeness (i.e. no unpleasant odour, visible signs of decay or degradation associated with spoilage). Emulating the traditional process used in Zambian home kitchens, each sample of dried Kapenta was dry roasted in batches in a large pan, and the four samples were combined into one 2-kg sample. This sample was taken to a small-scale miller at an open market for grinding into Kapenta dried fish powder. After grinding, the 2-kg sample of dried fish powder was shipped to the United States, where it was divided and distributed to two accredited commercial labs for analysis, including Mérieux NutriSciences and the Mississippi State Chemical Laboratory.

### Sensory panels: study design

**Fig. 2:**
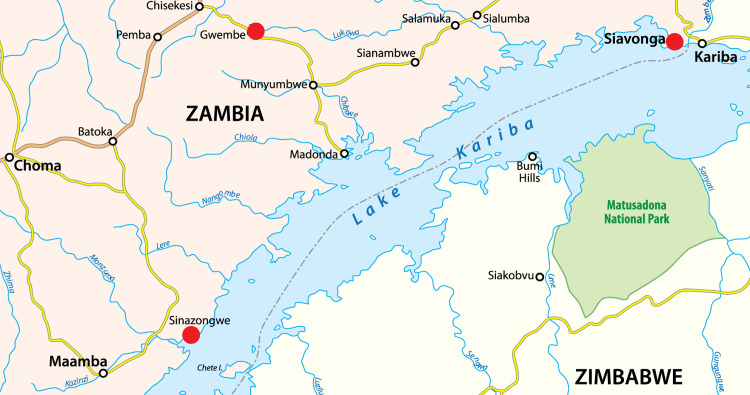
Map of Zambia’s Lake Kariba showing the village recruitment sites of Gwembe, Siavonga and Sinazongwe Districts

The study was conducted in the town of Siavonga, which is ∼195 km from the capital city of Lusaka and abuts the vast artificial water body of Lake Kariba (see Fig. 2). Caregivers were recruited from fishing villages in Gwembe, Siavonga and Sinazongwe Districts in Zambia’s Southern Province. The caregiver–child pairs convened in Siavonga and all caregivers were provided with childcare products (e.g. diapers, sanitary wipes and hand sanitiser) and transportation stipends to travel from their villages to the study site. Caregiver–child pairs from Gwembe and Sinazongwe were also provided with lodging at a Siavonga hotel for two nights as Gwembe and Sinazongwe are each more than 250 km from Siavonga.

#### Eligibility

To be eligible to participate in Sensory Panels I and II, women were required to self-reported that: (1) they were between 18 and 49 years of age; (2) they resided in Gwembe, Siavonga or Sinazongwe Districts; (3) they had no known allergy to dried fish powder, groundnut powder, orange-fleshed sweet potato, peanut/nut oil or vegetable oil; (4) their child was 6–23 months old; (5) their child had no known allergy to dried fish powder, groundnut powder, orange-fleshed sweet potato, peanut/nut oil, or vegetable oil; (6) their child had consumed fish and/or fish-based foods prior to recruitment into the study and (7) their child had consumed complementary maize porridge prior to recruitment into the study.

#### Preparatory phase

To prepare for Sensory Panels I and II, we conducted group interviews with women in rural fishing villages and other local experts in Zambia’s Southern Province to identify: (1) staple traditional dishes that were regularly consumed at the household level (including by infants and young children in the complementary feeding stage); (2) locally sourced ingredients that were commonly used in each of these staple traditional dishes and (3) whether dried fish powder was currently a common ingredient in these staple traditional dishes (including when the dishes were prepared as complementary foods for infants and young children). Based on this information, we chose three staple traditional dishes suitable to fortify with ComFA+Fish (i.e. chibwabwa fisashi, complementary maize porridge and bean-vegetable soup) and a novel dish (i.e. dried Kapenta chutney) whose primary ingredient is whole dried Kapenta. The four ComFA+Fish dishes that we developed use locally sourced Kapenta and other locally sourced staple ingredients (e.g. groundnut powder, orange-fleshed sweet potato powder and pumpkin leaf powder). In order to ensure that each ComFA+Fish dish could be produced at the household level, we pre-tested each recipe in Lusaka prior to implementing Sensory Panels I and II at the study site. During the pre-testing process, the ingredients for each dish were purchased at local markets, and a large batch of each dish was prepared in a standard Zambian kitchen using standard cooking tools (mortar and pestle, spoons, stove, etc.) and taste tested by the study team. The person who supervised preparation of the ComFA+Fish dishes during the pre-testing process also supervised preparation of the ComFA+Fish dishes at the study site. All ingredients used during Sensory Panels I and II were purchased at local markets at the study site.

#### Sensory panel I assessment

For Sensory Panel I, we developed a technical lexicon for use by caregivers to evaluate five sensory attributes and two additional attributes of the four ComFA+Fish dishes. Development of this lexicon and the panel’s procedures were informed by Madrelle *et al.* (2017) and others^(40–43)^ and the ComFA+Fish dishes that were assessed included: (1) fortified chibwabwa fisashi (a savory dish of dried dark green leafy vegetables); (2) savory Kapenta chutney (made from whole dried Kapenta cooked with tomato, onion, green pepper and spices); (3) fortified complementary maize porridge and (4) fortified bean-vegetable soup. Next, we simplified the technical definitions of all seven attributes, translated them from English into Tonga and pre-tested them among Tonga-speakers for clarity of language prior to use by the caregivers. For example, ‘distinct aromatic notes associated with the sample’ were simplified to ‘aroma or smell of the food’, and ‘distinct flavour notes associated with the sample’ were simplified to ‘flavour/taste of the food’.

For Sensory Panel I, caregivers were provided with a separate assessment form for each of the four ComFA+Fish dishes and were instructed to score the seven attributes of each dish based on the simplified lexicon using a five-point hedonic scale where 1 = extremely disliked and 5 = extremely liked. For this scale, the assessment form included black-and-white ‘smiley face’ emojis that correlated with each point of the five-point scale. Participants were instructed to mark the appropriate smiley face emoji with an ‘x’. All four ComFA+Fish dishes evaluated during Sensory Panel I were prepared onsite at separate cooking stations and served to the participants immediately. See Appendix 1 for the technical and simplified lexicons for caregivers.

#### Sensory panel II assessment

For Sensory Panel II, we developed a three-item set of descriptors for use by caregivers to assess their child’s global liking of ComFA+Fish complementary maize porridge (see Appendix 2). Development of these descriptors and the panel’s procedures were informed by Madrelle *et al.* (2017) and others^(40–43)^. We translated the three items from English into Tonga and pre-tested them among Tonga speakers for clarity of language prior to use by the caregivers. The fortified porridge was prepared onsite and served immediately to each caregivers, who fed the porridge to their child. Caregivers were instructed to feed the fortified porridge to their child during three consecutive tasting intervals (Times 1–3). The serving size of the fortified porridge was 50 g (∼8 oz.), and all children were fed their portion of the porridge using identical infant spoons and identical 8 oz. clear plastic cups. Caregivers were allowed to offer their child sips of bottled water between each of the three tasting intervals.

For item 1 of Sensory Panel II (see Appendix 3), we used the same set of black-and-white smiley face emojis used for Sensory Panel I. For this item, we instructed caregivers to assess their child’s global liking of the fortified porridge using a set of five descriptors of positive/negative behaviours exhibited by their child that correlated with a five-point hedonic scale where 1 = child extremely disliked the porridge and 5 = child extremely liked the porridge. For example, caregivers were instructed to indicate that their child extremely disliked the fortified porridge if the child ‘spit out the food, pushed the food away, vigorously turned their face away, stopped eating, became fussy, arched their back or pulled their body away when food was offered’. For item 1, caregivers were instructed to offer their child an initial set of three consecutive teaspoonfuls of the fortified porridge at Time 1 (T1), a second set of three teaspoonfuls of fortified porridge at Time 2 (T2) and a final set of three teaspoonfuls of fortified porridge at Time 3 (T3). After each tasting interval (T1, T2 and T3), caregivers were instructed to evaluate how much their child liked the fortified porridge using the five-point hedonic scale.

For item 2, we created black-and-white ‘bowl/portion’ images and caregivers were instructed to select the bowl/portion image that best correlated with the *actual* amount of fortified porridge that their child consumed during the entire meal (i.e. the sum of T1 + T2 + T3). This was assessed at the end of the meal, and caregivers were instructed to use a five-point hedonic scale where 1 = child consumed less than 1/4 of their portion and 5 = child consumed their entire portion. For item 2, the bowl/portion images correlated with each point of the five-point scale (see Appendix 3). For item 3, we used the same bowl/portion images used in item 2 and caregivers were instructed to select the bowl/portion icon that best correlated with the *relative* amount of fortified porridge that their child consumed during the entire meal (i.e. the sum of T1 + T2 + T3), as compared with their child’s regular intake of food during a meal served to the child at that time of day. This was assessed at the end of the meal, and caregivers used the same five-point hedonic scale and for item 3 as was used to answer item 2.

Item 4 was a ‘fill-in-the-blank’ item where caregivers provided the total number of servings of the fortified porridge that their child consumed. For item 4, caregivers were asked to sum the total number of full portions of fortified porridge that their child consumed during the entire meal (i.e. the sum of T1 + T2 + T3 *plus* any additional portions of the fortified porridge consumed after T3). This was assessed at the end of the meal, and the responses are disaggregated into the following categories in the results section: (1) consumed 3 full portions; (2) consumed 2 full portions; (3) consumed 1·5 full portions; (4) consumed 1 full portion and (5) consumed 1/2 portion. Item 4 also included a ‘comments’ section to provide caregivers with an opportunity to add any information that they felt might have influenced how their child responded to the fortified porridge during Sensory Panel II (i.e. whether their child was breast-fed right before or during the panel, etc.).

## Results

### Kapenta dried fish powder: nutrient composition results

For Table 1, ages were categorised into three groups according to dietary reference intakes (DRI), which includes infants aged 7–12 months, children aged 1–3 years and women aged 19–50 years^(44,45)^. Recommended DRI values for infants, children and women were obtained from the National Academies’ Institute of Medicine. DRI values include recommended dietary allowances and adequate intakes. Recommended dietary allowances are the level of intake of essential nutrients that are determined by the Food and Nutrition Board of the National Academies’ Institute of Medicine to be adequate to meet the known nutrient needs of practically all healthy persons^(44)^. Adequate intakes are used when there is not enough data to calculate an average requirement; these are the average nutrient levels consumed daily by a typical healthy population that is assumed to be adequate for the population’s needs^(ibid.)^.

**Table 1:** Dietary reference intakes (DRI) and percentages of DRI met for infants, children and women per serving of Kapenta dried fish powder

Nutrients	Kapenta dried fish powder (100 g prepared)	Infants 7–12 months		Children 1–3 years		Women 19–50 years	
		DRI value	% DRI met (10 g prepared)	DRI value	% DRI met (20 g prepared)	DRI value	% DRI met (30 g prepared)
Energy (kcal)	284·6	Variable		Variable		Variable	
Protein (g)	71·16	**11**	65	**13**	109	**46**	46
Total fat (g)	13·33	30*	4	–	–	–	–
*n* 3 fatty acids							
DHA (g)	9·19	–		–	–	–	–
DPA (g)	1·037	–		–	–	–	–
EPA (g)	5·13	–		–	–	–	–
Choline (mg)	365	150*	24	200*	37	425*	26
Minerals							
Ca (mg)	2860	260*	110	**700**	82	**1000**	86
Iodine (μg)	16	130*	1	**90**	4	**150**	3
Fe (mg)	10·6	**11**	10	**7**	30	**18**	18
Mg (mg)	158	75*	21	**80**	40	**320**	15
Potassium (mg)	1390	860*	16	2000*	14	2600*	16
Se (mg)	0·12	20*	–	**20**	–	**55**	–
Na (mg)	309	370*	8	800*	8	1500*	6
Zn (mg)	12·8	**3**	43	**3**	85	**8**	48
Vitamins							
Vitamin A (μg)	1260	500*	25	**300**	84	**700**	54
Vitamin B_12_ (μg)	11	0·5*	220	**0·9**	244	**2·4**	138
Vitamin D (μg)	42·6	10*	43	**15**	57	**15**	85
*α* tocopherol (μg)	1·3	5*	3	**6**	4	**15**	3

Note:

* This table presents recommended dietary allowances (RDA) in bold type and adequate intakes (AI) in ordinary type followed by an asterisk.

Recommendations regarding highest allowable mercury concentrations in fish were obtained from a joint advisory from the U.S. Food and Drug Administration and the U.S. Environmental Protection Agency^(46)^. The mercury concentration in the Kapenta dried fish powder was found to be 0·14 ug/100 g. This concentration is below the Food and Drug Administration/Environmental Protection Agency highest allowable average mercury concentration in fish per serving of 15 ug/100 g, when consuming three servings of fish/week^(ibid.)^.

The total amount of Kapenta dried fish powder used in each ComFA+Fish dish varied according to recipe. For example, the recipe for the fortified chibwabwa fisashi included 64 g of dried fish powder, the recipes for both the fortified complementary maize porridge and the fortified bean-vegetable soup included 128 g of dried fish powder and the recipe for the savory Kapenta chutney included 256 g of whole dried fish. The recommended serving sizes for each ComFA+Fish dish varied according to age category. For example, recommended serving sizes ranged from 8 to 10 g for infants aged 6–11-month-olds, 16–20 g for children aged 12–23-month-olds, 32–40 g for children aged 24 months and older and 32–40 g for adolescents and adults^(44,45)^. For Table 1, we calculated the percentage of DRI met for various nutrients, minerals and vitamins based on serving sizes of 10 g for infants aged 7–12 months, 20 g for children aged 1–3 years and 30 g for women aged 19–50 years.

As Table 1 indicates, the sample of Kapenta dried fish powder contained high percentages of the DRI for protein across all age categories and appreciable percentages of the DRI for choline across all age categories. In terms of protein, 10 g of the sample contained 65 % of the DRI for infants, 20 g contained 109 % of the DRI for children and 30 g contained 46 % of the DRI for women. In terms of choline, 10 g of the sample contained 24 % of the DRI for infants, 20 g contained 37 % of the DRI for children and 30 g contained 26 % of the DRI for women.

The sample contained high percentages of the DRI for Ca and Zn across all age categories, appreciable percentages of the DRI for Mg across all age categories and appreciable percentages of the DRI for Fe for children and women. In terms of Ca, 10 g of the sample contained 110 % of the DRI for infants, 20 g contained 82 % of the DRI for children and 30 g contained 86 % of the DRI for women. In terms of Zn, 10 g of the sample contained 43 % of the DRI for infants, 20 g contained 85 % of the DRI for children and 30 g contained 48 % of the DRI for women. In terms of Mg, 10 g of the sample contained 21 % of the DRI for infants, 20 g contained 40 % of the DRI for children and 30 g contained 15 % of the DRI for women. In terms of Fe, 20 g of the sample contained 30 % of the DRI for children and 30 g contained 18 % of the DRI for women.

The sample contained extremely high percentages of the DRI for vitamin B12 across all age categories and appreciable percentages of the DRI for vitamin A and vitamin D across all age categories. In terms of vitamin A, 10 g of the sample contained 25 % of the DRI for infants, 20 g contained 84 % of the DRI for children and 30 g contained 54 % of the DRI for women. In terms of vitamin B_12_, 10 g of the sample contained 220 % of the DRI for infants, 20 g contained 244 % of the DRI for children and 30 g contained 138 % of the DRI for women. In terms of vitamin D, 10 g of the sample contained 43 % of the DRI for infants, 20 g contained 57 % of the DRI for children and 30 g contained 85 % of the DRI for women.

### Descriptive statistics

As Table 2 indicates, an equal number of caregiver–child pairs were recruited from the districts of Gwembe, Siavonga and Sinazongwe (*n* 42 caregivers; *n* 42 children). Among the caregivers, 59·5 % were aged 19–29 years and 40·5 % were aged 30–44 years. Among the children, 38·1 % were aged 6–11 months and 61·9 % were aged 12–23 months.

**Table 2.** Demographics

	Gwembe District		Siavonga District		Sinazongwe District		Total	
	%	*n*	%	*n*	%	*n*	%	*n*
Caregivers’ district	33·3	14	33·3	14	33·3	14	100	42
Caregivers’ mean age (sd), Range: 19–44 years	26·2	5·8	24·8	5	31·5	6·7	27·5	6·4
Children’s mean age (sd), Range: 6–23 months	14	5	10·3	3·5	14·6	5·3	13	4·9
Male children	35·7	5	35·7	5	57·1	8	42·9	18
Female children	64·3	9	64·3	9	42·9	6	57·1	24

### Sensory panel I results: caregivers’ evaluation of four ComFA+Fish dishes

#### Comparison of attribute scores

Among the four ComFA+Fish dishes, the fortified chibwabwa fisashi was evaluated highest among caregivers, followed by the savory Kapenta chutney, the fortified complementary maize porridge and the fortified bean-vegetable soup. Below, we present the results for each of the four dishes derived from averaging the results of the five sensory attributes for each dish (i.e. aroma, appearance, mouthfeel/texture, flavour/taste and sweetness). The disaggregated results and the average of these results for each ComFA+Fish dish are presented in Tables 2–7. We next combined the averaged results for the two response categories of ‘extremely liked’ and ‘liked’ into one category of ‘extremely liked/liked,’ and this result is presented below for each ComFA+Fish dish. Finally, we separately present the results for the two additional attributes of convenience and overall acceptability for each ComFA+Fish dish.

**Table 3:** Sensory panel I: caregivers’ evaluation of ComFA+Fish chibwabwa fisashi (*n* 42)

Sensory attributes	Extremely liked		Liked		Neutral		Disliked		Extremely disliked	
	%	*n*	%	*n*	%	*n*	%	*n*	%	*n*
1. Aroma	92·9	39	4·8	2	–		–		2·4	1
2. Appearance	88·1	37	9·5	4	–		–		2·4	1
3. Mouth feel/texture	83·3	35	11·9	5	–		–		4·8	2
4. Flavour/taste	92·9	39	4·8	2	–		–		2·4	1
5. Sweetness	75	21	10·7	3	14·3	4	–		–	
Average of attributes 1–5	86·4	34	8·3	3	14·3	4	–		12	1
Non-sensory attributes										
6. Convenience	73·8	31	19	8	4·8	2	2·4	1	–	
7. Overall acceptability	95·2	40	4·8	2	–		–		–	

**Table 4:** Sensory panel I: caregivers’ evaluation of ComFA+Fish savory Kapenta chutney (*n* 42)

Sensory attributes	Extremely liked		Liked		Neutral		Disliked		Extremely disliked	
	%	*n*	%	*n*	%	*n*	%	*n*	%	*n*
1. Aroma	88·1	37	9·5	4	2·4	1	–	–	–	
2. Appearance	78·6	33	21·4	9	–		–	–	–	
3. Mouth feel/texture	90·5	38	4·8	2	2·4	1	–	–	2·4	1
4. Flavour/taste	95·2	40	2·4	1	2·4	1	–	–	–	
5. Sweetness	7·31	19	11·5	3	3·8	1	–	–	11·5	3
Average of attributes 1–5	71·9	33·4	9·9	3·8	2·2	0·8	–	–	2·8	0·8
Non-sensory attributes										
6. Convenience	85·7	36	7·1	3	4·8	2	–	–	2·4	1
7. Overall acceptability	97·6	41	2·4	1	–		–	–	–	

**Table 5:** Sensory panel I: caregivers’ evaluation of ComFA+Fish complementary maize porridge (*n* 41)

Sensory attributes	Extremely liked		Liked		Neutral		Disliked		Extremely disliked	
	%	*n*	%	*n*	%	*n*	%	*n*	%	*n*
1. Aroma	69	29	19	8	2·4	1	–		4·8	2
2. Appearance	69	29	23·8	10	–		–		2·4	1
3. Mouth feel/texture	59·5	25	19·5	8	2·4	1	–		14·6	6
4. Flavour/taste	54·8	23	34·1	14	2·4	1	–		2·4	1
5. Sweetness	52·4	22	14·3	6	9·5	4	–		7·1	3
Average of attributes 1–5	60·9	25·6	22·6	9·2	3·3	1·4	–		6·3	2·6
Non-sensory attributes										
6. Convenience	66·7	28	23·8	10	–		–		2·4	1
7. Overall acceptability	66·7	28	21·4	9	2·4	1	2·4	1	4·8	2

**Table 6:** Sensory panel I: caregivers’ evaluation of ComFA+Fish bean-vegetable soup (*n* 24)

Sensory attributes	Extremely liked		Liked		Neutral		Disliked		Extremely disliked	
	%	*n*	%	*n*	%	*n*	%	*n*	%	*n*
1. Aroma	56	14	16	4	12	3	4	1	12	3
2. Appearance	28·6	12	14·3	6	20·8	5	–		4·2	1
3. Mouth feel/texture	54·2	13	16·7	4	16·7	4	–		12·5	3
4. Flavour/taste	54·2	13	20·8	5	12·5	3	4·2	1	8·3	2
5. Sweetness	60	9	13·3	2	6·7	1	6·7	1	13·3	2
Average of attributes 1–5	50·6	12·2	16·2	4·2	13·7	3·2	2·9	0·6	10	2·2
Non-sensory attributes										
6. Convenience	62·5	15	25	6	4·2	1	–		8·3	2
7. Overall acceptability	54·2	13	33·3	8	8·3	2	–		4·2	1

**Table 7:** Sensory panel II: evaluation by caregivers of global liking, actual intake and relative intake of ComFA+Fish complementary maize porridge among 6–11-month-olds (*n* 16)

1. Child’s global liking at three tasting intervals:	Extremely liked		Liked		Neutral		Disliked		Extremely disliked	
	%	*n*	%	*n*	%	*n*	%	*n*	%	*n*
Time 1 (T1)	87·5	14	12·5	2	–		–		–	
Time 2 (T2)	87·5	14	12·5	2	–		–		–	
Time 3 (T3)	81·2	13	12·5	2	–		–		6·3	1
2. Child’s actual consumption:	Consumed entire portion		Consumed 3/4 portion		Consumed 1/2 portion		Consumed 1/4 portion		Consumed <1/4 portion	
T1 + T2 + T3	37·5	6	31·3	5	31·3	5	–		–	
3. Child’s relative consumption:	Consumed their regular amount		Consumed 3/4 of their regular amount		Consumed 1/2 of their regular amount		Consumed 1/4 of their regular amount		Consumed <1/4 of their regular amount	
T1 + T2 + T3 *v*. child’s regular intake	56·3	9	12·5	2	31·3	5	–		–	
4. Full portions child consumed:	Consumed 3 full portions		Consumed 2 full portions		Consumed 1·5 full portions		Consumed 1 full portion		Consumed 1/2 portion	
T1 + T2 + T3+ addtl. portions	–		6·3	1	–		81·2	13	12·5	2

##### Fortified chibwabwa fisashi: averaged sensory attribute scores

As Table 3 indicates, when caregivers’ (*n* 42) scores for the five sensory attributes of the fortified chibwabwa fisashi were averaged, 94·7 % of caregivers extremely liked/liked the sensory attributes of this dish. A majority of caregivers also extremely liked/liked the convenience (92·8 %) and overall acceptability (100 %) of the fortified chibwabwa fisashi.

##### Savory Kapenta chutney: averaged sensory attribute scores

As Table 4 indicates, when caregivers’ (*n* 42) scores for the five sensory attributes of the savory Kapenta chutney were averaged, 81·8 % of caregivers extremely liked/liked the sensory attributes of this dish. A majority of caregivers also extremely liked/liked the convenience (92·8 %) and overall acceptability (100 %) of the savory Kapenta chutney.

##### Fortified complementary maize porridge: averaged sensory attribute scores

As Table 5 indicates, when caregivers’ (*n* 41) scores for the five sensory attributes of the fortified complementary maize porridge were averaged, 83·5 % of caregivers extremely liked/liked the sensory attributes of this dish. A majority of caregivers also extremely liked/liked the convenience (90·5 %) and overall acceptability (88·1 %) of the fortified complementary maize porridge.

##### Fortified bean-vegetable soup: averaged sensory attribute scores

As Table 6 indicates, when caregivers’ (*n* 24) scores for the five sensory attributes of the fortified bean-vegetable soup were averaged, 66·8 % of caregivers extremely liked/liked the sensory attributes of this dish. A majority of caregivers also extremely liked/liked the convenience (87·5 %) and overall acceptability (87·5 %) of the fortified bean-vegetable soup.

### Sensory panel II results: caregivers’ evaluation of their child’s global liking, actual intake and relative intake of ComFA+Fish complementary maize porridge

#### Global liking results among infants aged 6–11 months

As Table 7 indicates, an average of 85·4 % of caregivers (*n* 16) reported that their 6–11-month-olds extremely liked the fortified porridge at Time 1 (T1), Time 2 (T2) and Time (T3) and these scores ranged from 87·5 % (T1, T2) to 81·2 % (T3). An average of 12·5 % of caregivers reported that their 6–11-month-olds liked the fortified porridge at T1 + T2 + T3, and these scores did not vary across T1, T2 and T3.

#### Actual fortified porridge intake among infants aged 6–11 months

In terms of actual porridge intake, a majority of caregivers (68·8 %) reported that their 6–11-month-olds consumed 3/4 or more of their portion of fortified porridge at T1 + T2 + T3. In contrast, 31·3 % of caregivers reported that their 6–11-month-olds consumed 1/2 of their portion of fortified porridge at T1 + T2 + T3.

#### Relative fortified porridge intake among infants aged 6–11 months

In terms of relative porridge intake, a majority of caregivers (68·8 %) reported that their 6–11-month-olds consumed 3/4 or more of their regular amount of food at T1 + T2 + T3 as compared with their child’s regular intake of food during a meal served to the child at that time of day. In contrast, 31·3 % of caregivers reported that their 6–11-month-olds consumed 1/2 of their regular amount of food at T1 + T2 + T3 as compared with their child’s regular intake of food during a meal served to the child at that time of day. Finally, a majority of caregivers (87·5 %) reported that their 6–11-month-olds consumed one or more full portions of the fortified porridge (i.e. T1 + T2 + T3 plus any additional portions of the fortified porridge consumed after T3).

#### Global liking results among children aged 12–23 months

As Table 8 indicates, an average of 85·4 % of caregivers (*n* 26) reported that their 12–23-month-olds extremely liked the fortified porridge at T1 + T2 + T3 and these scores ranged from 88·5 % (T2, T3) to 84·6 % (T1). An average of 14·1 % of caregivers reported that their 12–23-month-olds liked the fortified porridge at T1 + T2 + T3 and these scores ranged from 7·7 % (T1) to 3·8 % (T2, T3).

**Table 8:** Sensory panel II: evaluation by caregivers of global liking, actual intake and relative intake of ComFA+Fish complementary maize porridge among 12–23-month-olds (*n* 26)

1. Child’s global liking at three tasting intervals:	Extremely liked		Liked		Neutral		Disliked		Extremely disliked	
	%	*n*	%	*n*	%	*n*	%	*n*	%	*n*
Time 1 (T1)	84·6	22	7·7	2	–		–		7·7	2
Time 2 (T2)	88·5	23	3·8	1	–		–		7·7	2
Time 3 (T3)	88·5	23	3·8	1	–		–		7·7	2
2. Child’s actual consumption:	Consumed entire portion		Consumed 3/4 portion		Consumed 1/2 portion		Consumed 1/4 portion		Consumed <1/4 portion	
T1 + T2 + T3	80·7	21	3·8	1	3·8	1	7·7	2	3·8	1
3. Child’s relative consumption:	Consumed their regular amount		Consumed 3/4 of their regular amount		Consumed 1/2 of their regular amount		Consumed 1/4 of their regular amount		Consumed <1/4 of their regular amount	
T1 + T2 + T3 *v*. child’s regular intake	88·5	23	7·7	2	–		–		3·8	1
4. Full portions child consumed:	Consumed 3 full portions		Consumed 2 full portions		Consumed 1·5 full portions		Consumed 1 full portion		Consumed 1/2 portion	
T1 + T2 + T3+ addtl. portions	3·9	1	42·3	11	3·9	1	50	13	–	

#### Actual fortified porridge intake among children aged 12–23 months

In terms of actual porridge intake, a majority of caregivers (84·5 %) reported that their 12–23-month-olds consumed 3/4 or more of their portion of fortified porridge at T1 + T2 + T3. In contrast, 3·8 % of caregivers reported that their 12–23-month-olds consumed 1/2 of their portion of fortified porridge at T1 + T2 + T3.

#### Relative fortified porridge intake among children aged 12–23 months

In terms of relative porridge intake, a majority of caregivers (96·2 %) reported that their 12–23-month-olds consumed 3/4 or more of their regular amount of food at T1 + T2 + T3. In contrast, 3·8 % of caregivers reported that their 12–23-month-olds consumed less than 1/4 of their regular amount of food at T1 + T2 + T3. Finally, all caregivers (100 %) reported that their 12–23-month-olds consumed one or more full portions of the fortified porridge (i.e. T1 + T2 + T3 plus any additional portions of the fortified porridge consumed after T3).

## Discussion

### Sensory panel I: sensory attribute results among caregivers

The sensory results for the four ComFA+Fish dishes assessed during Sensory Panel I are promising, given that a fish-based protein/micronutrient blend such as ComFA+Fish has the potential to add an unusual or unexpected aroma, flavor, etc., to a familiar dish that could negatively impact the acceptability of one or more of that dish’s sensory attributes. The averaged sensory attribute scores for three of the four ComFA+Fish dishes indicate high acceptability among caregivers, with averaged ‘extremely liked/liked’ scores that ranged from nearly 95 % (fortified chibwabwa fisashi) to nearly 82 % (savory Kapenta chutney). The averaged sensory attribute score for the fortified bean-vegetable soup indicates that it was less acceptable in comparison to the other three ComFA+Fis dishes. For example, although nearly 67 % of caregivers extremely liked/liked the averaged sensory attributes of the fortified bean-vegetable soup, nearly 14 % were neutral and nearly 13 % extremely disliked/disliked the dish’s sensory attributes.

When the disaggregated scores are examined, the appearance of the fortified bean-vegetable soup was assessed as less desirable than the appearance of the other three ComFA+Fish dishes. For example, nearly 43 % of caregivers extremely liked/liked the soup’s appearance, while nearly 21 % of caregivers were neutral regarding the soup’s appearance. In contrast, an overwhelming majority of caregivers extremely liked/liked the appearance of the fortified chibwabwa fisashi (∼98 %), savory Kapenta chutney (100 %) and fortified complementary maize porridge (∼93 %), and no caregivers were neutral regarding the appearance of these ComFA+Fish dishes. These results suggest that the recipe for the fortified bean-vegetable soup should be adjusted to improve appearance.

### Sensory panel I: non-sensory attribute results among caregivers

The results suggest high acceptability among caregivers of the attributes of convenience and of overall acceptability for all four ComFA+Fish dishes. A majority of caregivers extremely liked/liked the convenience of all four of the ComFA+Fish dishes, and a majority of caregivers scored the overall acceptability as high for all four of the ComFA+Fish dishes. Given that the extreme ‘time poverty’ of resource-limited women in LMIC is well documented^(47–49)^, the attribute of convenience is key to scaling a protein/micronutrient blend such as ComFA+Fish and ensuring that resource-limited women: (1) adopt the product long-term and (2) use it on a regular basis (i.e. in at least one meal/d for children aged 6–23 months). A nutrient-dense supplement that does not account for the extreme time poverty of female caregivers (i.e. does not ensure that the product is easy-to-use on a daily basis) will likely encounter barriers to widespread adoption regardless of its efficacy. Therefore, ComFA+Fish is designed with convenience as a central attribute. By utilising dried fish powder as its primary ingredient, ComFA+Fish is a nutrient-dense, locally accessible product that can easily be added to traditional dishes that family members are already used to consuming, including infants and young children who are in the complementary feeding stage. It is expected that this approach also helped ensure that the ComFA+Fish dishes received high scores for overall acceptability, as this is an attribute that Puri *et al.* (2022) argue is more important ‘from the consumer point of view’ than are individual sensory attributes^(41,5)^.

### Sensory panel II: evaluation by caregivers of global liking among infants and young children

The Sensory Panel II results indicated that the ComFA+Fish complementary maize porridge was evaluated among the majority of caregivers as highly acceptable to both 6–11-month-olds and 12–23-month-olds. These results are promising given that traditional (non-fortified) maize porridge is arguably the most commonly consumed complementary food among infants and young children in Zambia and many other LMIC across sub-Saharan Africa^(37,38)^. That a dried fish powder-based protein/micronutrient blend such as ComFA+Fish might not be appetising to infants and young children when added to complementary maize porridge was of critical concern in terms of scalability. These results suggest that fortifying complementary maize porridge with ComFA+Fish did not have a negative impact on infants’ and young children’s food intake. This is a promising result, given that infants’ and young children’s gastric capacity can only accommodate small meals and, therefore, every meal should be nutrient dense.

The present study’s results should be interpreted within the limitations of a pilot study that included a relatively small sample recruited using a convenience sampling strategy. Since ComFA+Fish is an innovative protein/micronutrient blend and the study is the first to evaluate its sensory and other attributes, there is currently no reference to which to compare our results. Social desirability bias might have affected caregivers’ responses. For example, it is important to note that only twenty-four of forty-two caregivers completed their evaluation of the fortified bean-vegetable soup, and their responses for the soup’s sensory attributes were not as positive as were caregivers’ responses for the sensory attributes of the other three ComFA+Fish dishes. These results suggest that the recipe for the fortified bean-vegetable soup will need to be modified in order to increase its sensory attribute scores. Further, the level of non-responses for the sensory attribute of sweetness may have skewed the averaged results. For example, while thirty-five of forty-two caregivers scored the attribute of sweetness for the fortified complementary maize porridge, twenty-eight of forty-two caregivers scored this attribute for the fortified chibwabwa fisashi, twenty-six of forty-two caregivers scored this attribute for the savory Kapenta chutney and fifteen of twenty-four caregivers scored this attribute for the fortified bean-vegetable soup. These results suggest that this attribute’s definition should be clarified and revised for future sensory panels.

### Conclusion

The present study contributes to the growing body of evidence that the consumption of aquatic animal-source foods including locally sourced dried fish powder are promising as ways to reduce stunting and anemia^(15,17–19,50)^. The results suggest that ComFA+Fish is strategically well placed to fill nutritional gaps to address micronutrient deficiencies among vulnerable 6–23-month-olds, pregnant/lactating women and other household members (e.g. adolescent girls) in Zambia and other LMIC. That all four ComFA+Fish dishes received high scores for the attribute of convenience suggests that this protein/micronutrient blend has the potential for wide scale adoption for daily fortification of complementary maize porridge and other traditional dishes consumed in Zambia and across sub-Saharan Africa.

A second advantage of ComFA+Fish – whose primary ingredient is whole pelagic small fish that are dried and ground into a fine powder – is that cold-storage methods of preserving fresh fish are rarely available to small-scale men and women fishers and processors in LMIC and, as a consequence, fresh fish can be lost through spoilage before they can be sold or processed. Pelagic small fish are easily dried whole, which not only concentrates their nutritional density but prevents spoilage. A third advantage of ComFA+Fish is that it utilises widely available local ingredients (e.g. dried fish powder, dried pumpkin leaves, etc.) and, therefore, can be produced at the household-level using common kitchen tools and is also commercially produced with a mechanised grinder for sale in large batches or as affordable sachets in local markets^(15,17–19,39)^. Because the key ingredient of ComFA+Fish is locally sourced dried fish powder, it has the potential to allow wider access to affordable and palatable sources of protein and micronutrients for urban and rural populations living in extreme poverty in Zambia and across sub-Saharan Africa for whom the lack of dietary diversity and reliance on high-phytate, low-protein maize-based diets increases their vulnerability to hidden hunger.

What makes the results promising is that – rather than trying to introduce a new nutrient-dense product to market that would likely not be accessible or affordable for the urban and rural poor – ComFA+Fish is a nutrient-dense product whose primary ingredient is an aquatic animal-sourced food (whole pelagic small fish ground into a fine powder) that is widely accessible, affordable and is both commonly produced at-home and purchased as a pre-ground powder in urban and rural local markets. Because ComFA+Fish uses locally sourced ingredients that already have high acceptability among our target population of vulnerable urban and rural households, scaling ComFA+Fish across different regions and countries will leverage the familiarity of local foods.

Having determined the acceptability of four ComFA+Fish dishes, our next steps are to: (1) complete a 6-month shelf-life study of Kapenta dried fish powder (underway); (2) as needed, adjust the amount of dried fish powder per recipe to ensure that the recommended serving size per age group (e.g. 10 g for infants aged 7–12 months) meets the recommended DRI values for infants, children and women without compromising convenience, overall acceptability and palatability and (3) collaborate with tiered in-country scaling partners to scale ComFA+Fish at the national (e.g. school feeding programs), regional (mid-level entrepreneurs) and village levels (micro-enterprises and households) across Zambia and sub-Saharan Africa.

## Supporting information

Ragsdale et al. supplementary material Ragsdale et al. supplementary material

## Supplementary material

For supplementary material accompanying this paper visit https://doi.org/10.1017/S1368980023002586
