# Supplementary material for: Sensory panel results of a dried fish powder supplement among caregivers and young children in Zambia
Source: Public Health Nutr. 2023 Nov 30;27(1):e32. doi: 10.1017/S1368980023002586 (PMC10897570; doi:10.1017/S1368980023002586)
Supplement: Ragsdale et al. supplementary material [file S1368980023002586sup001.docx]

| **Appendix 1.** Sensory Panel I Assessment: Technical and simplified lexicons for use by caregivers to assess seven attributes of four ComFA+Fish-fortified dishes. | | | |
| --- | --- | --- | --- |
| **Descriptor** | **Technical**  **Definition** | **Simplified**  **Definition** | **Scoring** |
| 1. Aroma | Distinct aromatic notes associated with the sample | Aroma/smell of the food | 1=Disliked very much  2=Disliked  3=Neither liked nor disliked  4=Liked  5=Liked very much |
| 1. Appearance | Color: Description of predominant color of the sample and its relative intensity | Main color of the food | 1=Disliked very much  2=Disliked  3=Neither liked nor disliked  4=Liked  5=Liked very much |
| 1. Texture/ Mouth Feel | Smoothness: Geometrical attribute associated with the overall degree of absence of particles within the sample | Overall smoothness of the food and lack of any grainy texture or particles | 1=Disliked very much  2=Disliked  3=Neither liked nor disliked  4=Liked  5=Liked very much |
| 1. Flavor/Taste | Distinct flavor notes associated with the sample | Flavor/taste of the food | 1=Disliked very much  2=Disliked  3=Neither liked nor disliked  4=Liked  5=Liked very much |
| 1. Sweetness | Gustatory sensation that remains after swallowing that is associated with sample’s overall sweetness as compared to boiled sweet potato | Lingering taste after swallowing food that is similar to the sweet taste of boiled sweet potato | 1=Disliked very much  2=Disliked  3=Neither liked nor disliked  4=Liked  5= Liked very much |
| 1. Convenience | How well you like or disklike how easy it will be to use at least once per day in a complementary meal for 6-23 month-olds | How well you like or disklike how easy it will be to use at least once per day in a meal for your 6-23 month-old | 1=Disliked very much  2=Disliked  3=Neither liked nor disliked  4=Liked  5=Liked very much |
| 1. Overall Acceptability | How well you like or dislike the sample when you consider its smell, appearance, texture, flavor, sweetness, and how convenient it will be to use at least once per day in a meal for 6-23 month-olds | How well you like or dislike the food’s smell, appearance, texture, flavor, sweetness, and how easy it will be to use at least once per day in a meal for your 6-23 month-old | 1=Disliked very much  2=Disliked  3=Neither liked nor disliked  4=Liked  5=Liked very much |

| **Appendix 2.** Sensory Panel II Assessment: Descriptors for use by caregivers to assess their child’s global liking of ComFA+Fish complementary maize porridge. | |
| --- | --- |
| **Item Descriptor** | **Scoring** |
| 1. Evaluation by caregivers of child’s ‘global liking’ of ComFA+Fish-fortified complementary porridge at three consecutive time intervals (T1, T2, T3). |  |
| 1. Child spit out the food, pushed the food away, vigorously turned their face away, stopped eating, became fussy, arched their back, pulled their body away when food was offered | 1=Child extremely disliked porridge |
| 1. Child looked away, frowned, wrinkled their nose when food was offered | 2=Child disliked porridge |
| 1. Child did not display overtly enthusiastic or negative reactions when food was offered | 3=Child neither liked nor disliked porridge (neutral) |
| 1. Child ate with little or no coaxing when food was offered, leaned forward, displayed a relaxed face | 4=Child liked porridge |
| 1. Child grabbed at the spoon to force it into their mouth when food was offered, displayed an excited face, made specific sounds of pleasure, ate enthusiastically, gestured enthusiastically with their arms and legs | 5=Child extremely liked porridge |
| 2. Estimate by caregivers of their child’s actual intake of ComFA+Fish-fortified complementary maize porridge at T1+T2+T3 (assessed at end of meal) | 1=Child consumed ˂1/4 of the portion  2=Child consumed 1/4 of the portion  3=Child consumed 1/2 of the portion  4=Child consumed 3/4 of the portion  5=Child consumed entire portion |
| 3. Estimate by caregivers of their child’s relative intake of ComFA+Fish-fortified complementary maize porridge at T1+T2+T3 as compared to their child’s regular intake of food during a meal at this time of day (assessed at end of meal) | 1=Child consumed ˂1/4 of their regular amount  2=Child consumed 1/4 of their regular amount  3=Child consumed 1/2 of their regular amount  4=Child consumed 3/4 of their regular amount  5=Child consumed their regular amount |
| 4. Total number of servings child consumed [Fill in the blank]: ____  Describe any feeding situation (e.g., Was child was breastfed right before taste-test?...): ________ |  |

**Appendix 3.** Sensory Panel II Scoring Sheet: Smiley face emojis, bowl/portion images, and descriptive text used by caregivers to assess their child’s response to the ComFA+Fish Complementary Maize Porridge (English version).
